# Feasibility of Recombinant Human TSH as a Preparation for Radioiodine Therapy in Patients with Distant Metastases from Papillary Thyroid Cancer: Comparison of Long-Term Survival Outcomes with Thyroid Hormone Withdrawal

**DOI:** 10.3390/diagnostics12010221

**Published:** 2022-01-17

**Authors:** Hsi-Chen Tsai, Kung-Chu Ho, Shih-Hsin Chen, Jing-Ren Tseng, Lan-Yan Yang, Kun-Ju Lin, Ju-Chin Cheng, Miaw-Jene Liou

**Affiliations:** 1 Department of Nuclear Medicine, New Taipei Municipal TuCheng Hospital (Built and Operated by Chang Gung Medical Foundation), New Taipei 236, Taiwan; toughprimenumber@gmail.com (H.-C.T.); drtsengjr@gmail.com (J.-R.T.); 2 Department of Nuclear Medicine, Linkou Chang Gung Memorial Hospital, Taoyuan 333, Taiwan; b8301068@gmail.com; 3 Department of Nuclear Medicine, Keelung Chang Gung Memorial Hospital, Keelung 204, Taiwan; yevgenyc@cgmh.org.tw (S.-H.C.); regina4651@gmail.com (J.-C.C.); 4 Biostatistics Unit, Clinical Trial Center, Chang Gung Memorial Hospital, Taoyuan 333, Taiwan; lyyang0111@gmail.com; 5 Department of Metabolism and Endocrinology, Linkou Chang Gung Memorial Hospital, Taoyuan 333, Taiwan

**Keywords:** papillary thyroid cancer, distant metastasis, radioiodine therapy, thyroid hormone withdrawal, recombinant human TSH stimulation

## Abstract

Background: this study was designed to compare the long-term survival outcomes of patients prepared for radioiodine (RAI) therapy using either thyroid hormone withdrawal (THW) or recombinant human thyrotropin (rhTSH) stimulation, by specifically focusing on cases with distant metastases from papillary thyroid cancer (PTC). Methods: A retrospective analysis was performed on 88 patients with distant metastases from PTC. Fifty-one and thirty-seven patients were prepared for RAI treatment by either THW or rhTSH stimulation, respectively. The primary endpoints were progression-free survival (PFS) and disease-specific survival (DSS). Results: The 10-year DSS rates of patients prepared for RAI therapy using either THW or rhTSH stimulation were 62.2% and 73.3%, respectively. Using multivariate analysis, RAI-avid metastases (*p* = 0.025) and preparation with rhTSH (*p* = 0.041) were identified as independent prognostic factors for PFS. Notably, PFS in the group of patients with RAI-avid metastases and preparation with rhTSH was significantly better than that in the other groups (*p* = 0.025). Conclusions: Preparation for RAI therapy using rhTSH stimulation is not inferior to THW preparation in terms of long-term survival outcomes experienced by patients with PTC and distant metastasis. Patients with RAI-avid metastases and preparation with rhTSH had the most favorable PFS.

## 1. Introduction

In the course of receiving radioiodine (RAI) therapy for patients without distant metastasis from differentiated thyroid cancer (DTC), recombinant human thyrotropin (rhTSH) has become an alternative modality of preparation for increasing thyrotropin (TSH), compared to thyroid hormone withdrawal (THW). Accumulating evidence indicates that rhTSH can avoid THW-induced severe hypothyroidism and an associated decrease in quality of life [1]. Further, patients treated by rhTSH stimulation may clear circulatory RAI radioactivity faster, resulting in less whole-body absorbed doses compared to THW [2]. Because of the relatively short-lived increase in serum TSH levels, rhTSH administration can also inhibit tumor growth [3]. Therefore, preparation for RAI with rhTSH is a safe and effective alternative to THW for patients with low- and intermediate-risk DTC [4] with comparable remnant ablation rates [5]. However, rhTSH stimulation is not currently approved by the American Thyroid Association as a preparation method for RAI adjunctive therapy in DTC patients with distant metastases [4].

Several studies of DTC patients with distant metastases [6,7,8,9] have shown that rhTSH offers a viable alternative to THW [6], with similar rates of tumor response and progression-free survival [8]. Another report found no significant difference between the two preparation methods in terms of 5-year overall survival rates in patients with RAI-avid distant metastases [9]. However, whether THW and rhTSH stimulation are equally effective in patients with RAI-avid distant metastases from DTC is a matter of ongoing debate. While some authors have advocated their equivalence [7], others demonstrated lower RAI uptake in distant metastases among patients who received rhTSH [10,11,12,13].

Papillary thyroid carcinoma (PTC) is the most common form of DTC [14]. The mortality rate of thyroid cancer has recently increased substantially, largely driven by higher incidence and mortality rates in patients with distant metastases from PTC [15]. In light of this worrying trend, this patient group is ideal for studying the long-term survival impact of preparation for RAI therapy with either THW or rhTSH stimulation. This research was, therefore, undertaken to specifically address this issue in patients with PTC. The evidence from this study can shed more light on the appropriateness of the current guidelines that argue against the use of rhTSH stimulation in patients with distant metastases from PTC.

## 2. Materials and Methods

### 2.1. Patients

We retrospectively reviewed the clinical charts of all patients with pathologically proven PTC who were referred to the Department of Nuclear Medicine at Chang Gung Memorial Hospital between January 2007 and December 2018. Patients with distant metastases from PTC who had undergone total thyroidectomy and had been prepared for high-dose (>30 mCi) RAI therapy using either THW or rhTSH stimulation were eligible. Distant metastases were defined as the spread of PTC to distant organs confirmed by biopsy or imaging investigations, including ^131^I whole-body scan (WBS), computed tomography (CT), magnetic resonance imaging (MRI), or positron emission tomography (PET). Exclusion criteria were as follows: (1) presence of thyroid tumors different from PTC; (2) missing data (e.g., no information on the date of diagnosis or distant metastases); (3) treatment with thyroid lobectomy or subtotal thyroidectomy; (4) loss to follow-up. Among 106 potentially eligible subjects, 18 cases met the exclusion criteria; therefore, a total of 88 patients were examined. Patients were staged according to the Eighth Edition of the American Joint Committee on Cancer (AJCC) Cancer Staging Manual [16].

### 2.2. Treatment Protocol

Patients were treated with total thyroidectomy—either with or without neck dissection—followed by thyroid remnant ablation with RAI (30–200 mCi; dose selected according to disease severity [4]). All participants had regular imaging and laboratory follow-up to detect possible persistent, recurrent, or metastatic disease. Surveillance was performed with CT, diagnostic ^131^I WBS, and measurement of serum thyroglobulin (Tg) values. Following lesion identification, patients underwent preparation to raise endogenous TSH levels and subsequently received additional doses of RAI (30–100 mCi and 100–200 mCi for regional lesions and distant metastasis, respectively). Whole-body scans were performed 6–8 days later to investigate RAI uptake. Patients with non-RAI-avid distant metastases underwent targeted therapy with sorafenib (starting from 2014) or lenvatinib (starting from 2016) at the physician’s discretion.

### 2.3. Preparation for RAI Treatment

All participants were provided with information on low-iodine diets and encouraged to adhere to them strictly for two weeks. The choice of preparation (THW vs. rhTSH) was based on a consensus between the patient and the physician in the absence of predefined criteria favoring an option. Patients in the THW group were withdrawn from long-acting levothyroxine for four weeks before receiving RAI (70–200 mCi). Patients in the rhTSH group were given two intramuscular injections of rhTSH (1.1 mg) on days 1 and 2. On day 3, they were orally administered RAI (70–200 mCi). In both groups, whole-body scans were performed 6–8 days later. Patients with new uptake foci on follow-up imaging were considered to have progressive disease.

### 2.4. Statistical Analysis

The primary study endpoints were progression-free survival (PFS) and disease-specific survival (DSS) of patients prepared with either THW or rhTSH stimulation. PFS and DSS curves from the date of distant metastasis to the date of disease progression or death of disease were estimated using the Kaplan–Meier method and compared with the log-rank test. Categorical variables were expressed as counts (percentages) and analyzed with the Fisher’s exact test. The associations between prognostic factors and survival endpoints were evaluated using univariate and multivariate Cox regression analyses. A backward stepwise selection procedure based on the probability of the Wald statistic was applied to select independent risk factors for survival outcomes. Results were expressed as hazard ratios (HRs) with 95% confidence intervals (CIs). Separate subgroup analyses of PFS were conducted based on selected independent risk factors identified by multivariate analysis. Statistical calculations were performed using SPSS, version 21.0 (IBM, Armonk, NY, USA). All tests were two-sided, and statistical significance was set as a *p*-value of <0.05.

## 3. Results

### 3.1. Patients

We identified 88 patients with distant metastases from PTC (59% women; mean age at diagnosis: 46.1 years). Distant metastases were diagnosed either at initial presentation (*n* = 44; 50%) or as distant recurrent lesions (*n* = 44; 50%). Isolated pulmonary metastases were present in 70% of the patients, followed by isolated bone metastases (15%), simultaneous pulmonary and bone metastases (13%), and metastases to other distant sites (2%). The participants were followed up for a median of 6.5 years (range: 1.0–18.1 years). Overall, 41 patients developed disease progression and 21 died at follow-up (two of other causes and 19 of PTC with distant metastases, with these deaths being used to determine DSS). Fifty-one and thirty-seven patients were prepared for RAI treatment by either THW or rhTSH stimulation, respectively. The general characteristics of the two study groups are presented in Table 1. There were no intergroup differences in terms of age at diagnosis, sex, site and diagnostic time of distant metastases, stage at diagnosis, cycles of RAI therapy, RAI avidity, and use of targeted therapy.

### 3.2. Long-Term Survival Impact of THW or rhTSH Preparation

**Figure 1:**
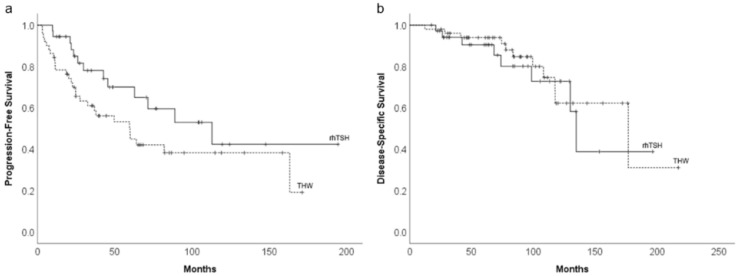
Kaplan–Meier plots of progression-free survival (PFS) (**a**) and disease-specific survival (DSS) (**b**) in patients prepared for RAI treatment by either THW (*n* = 51) or rhTSH stimulation (*n* = 37). There was no significant difference in terms of PFS and DSS between the two study groups.

Twenty-eight patients in the THW group and thirteen patients in the rhTSH group showed disease progression. Eleven patients in the THW group and eight in the rhTSH group died of the disease. There was no significant difference in terms of PFS and DSS between the two groups. The 5- and 10-year PFS rates in the THW and rhTSH groups were 47.7% vs. 71.0% and 38.3% vs. 43.5%, respectively (Figure 1a). There was no statistically significant difference in terms of DSS between the two groups. The 5-, 10- and 15-year DSS rates in the THW and rhTSH groups were 94.0% vs. 90.8%, 62.2% vs. 73.3%, and 31.1% vs. 39.1%, respectively (Figure 1b).

### 3.3. Risk Factors for PFS

**Figure 2:**
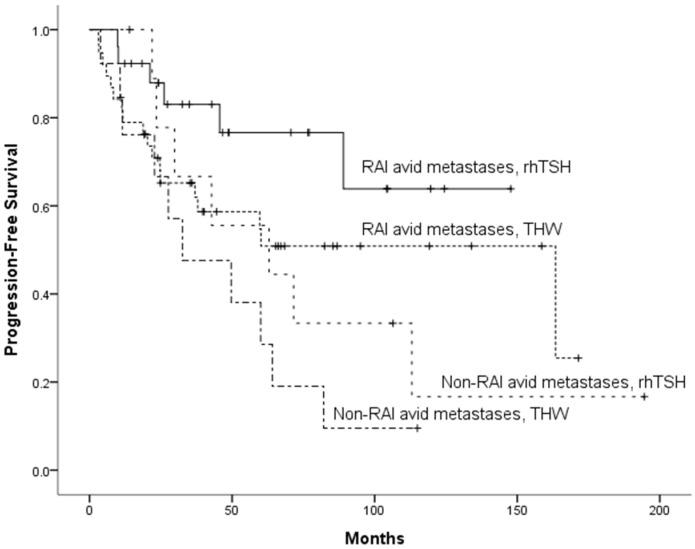
Kaplan–Meier plots of progression-free survival (PFS) in patients stratified according to RAI avidity and preparation method for RAI treatment (*p* = 0.025). The highest 5- and 10-year PFS rates were observed in patients with RAI-avid metastases who were prepared for RAI with rhTSH.

**Figure 3:**
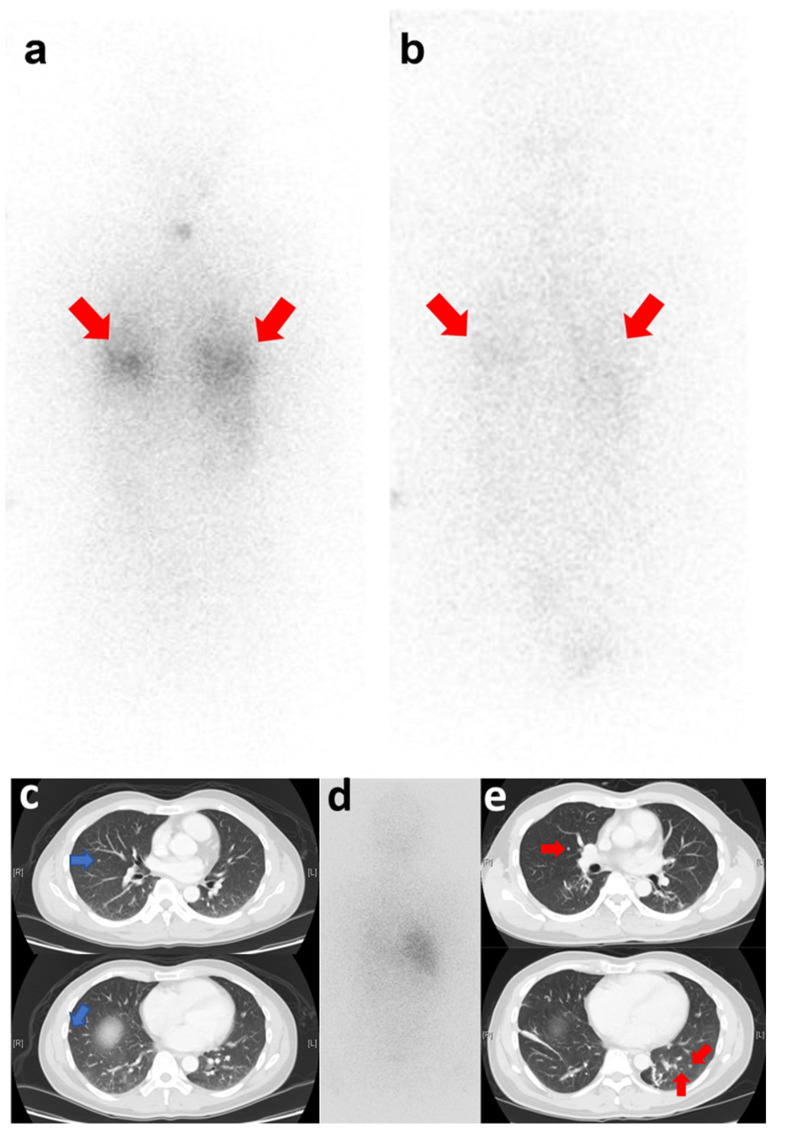
Posttreatment (100–150 mCi ^131^I) whole-body scan (WBS) images (posterior view) obtained from two patients in group 1 (**a**,**b**) and group 4 (**c**–**e**). (**a**) A 30-year-old woman prepared using rhTSH stimulation. Bilateral lung metastases (red arrows) were initially identified in 2015. (**b**) Three years later and following six treatment courses, regression of metastases was observed (red arrows) along with reduced ^131^I uptake. (**c**,**d**) A 34-year-old man prepared using THW. Right lung metastases were initially identified on computed tomography (CT) (blue arrows) in 2014, but no radioiodine avidity was evident on WBS images. (**e**) After three years and following three treatment courses, a CT scan revealed disease progression with bilateral lung metastases (red arrows).

As shown in our univariate analysis (Table 2), no significant risk factor for PFS was identified. After adjusting for potential confounders in our multivariate analysis, non-RAI-avid metastases (*p* = 0.025) and preparation with THW (*p* = 0.041) were selected in the model as independent adverse risk factors for PFS. For further stratification, the study patients were divided into four categories, as follows: RAI-avid metastases and preparation with rhTSH (group 1); RAI-avid metastases and preparation with THW (group 2); non-RAI-avid metastases and preparation with rhTSH (group 3); non-RAI-avid metastases and preparation with THW (group 4). The 5- and 10-year PFS rates of patients in groups 1, 2, 3, and 4 were 77.8%, 54.8%, 55.6%, and 28.6%, and 64.8%, 50.9%, 16.7%, and 9.5%, respectively (*p* = 0.025; Figure 2). Group 1 was a low-risk group (Figure 3a,b), whereas groups 2 and 3 were at intermediate risk (HR = 2.26; 95% CI: 0.90–5.72 and HR = 2.67; 95% CI: 0.89–7.99, respectively). The patients in group 4 were at high risk (HR = 4.42; 95% CI: 1.60–12.19, Figure 3c–e).

### 3.4. Risk Factors for DSS

The results of the univariate analysis (Table 3) revealed that the presence of stage I/II at diagnosis (*p* = 0.020), male sex (*p* = 0.015), and age at diagnosis ≥55 years (*p* = 0.003) were significantly associated with less favorable DSS. From the multivariate analysis, age at diagnosis (*p* = 0.003), the site of distant metastasis (*p* = 0.047), and sex (*p* = 0.017) were retained in the model as independent risk factors for DSS.

## 4. Discussion

The present research compared the long-term survival rates of patients with distant metastases from PTC prepared for RAI therapy with either THW or rhTSH stimulation. There are two main findings from the current study. First, preparation with rhTSH stimulation is not inferior to THW preparation in terms of DSS. Second, the preparation method was identified as being independently associated with PFS. Specifically, we found that patients with RAI-avid metastases who were prepared with rhTSH had the most favorable long-term PFS. Taken together, these results indicate that preparation with rhTSH does not have an adverse effect on long-term DSS, and may even be superior to traditional THW in the prevention of disease progression.

A previous study from our institution demonstrated that patients with distant metastases from PTC treated with high-dose radioactive iodine have 5-, 10-, and 20-year DSS rates of 88.6%, 68.1%, and 24.9%, respectively [17]. Although the authors did not specifically analyze DSS in relation to the preparation method for RAI, the survival figures are consistent with those observed in the current investigation. The independent adverse prognostic significance of older age and distant metastases to sites other than the lung that we observed in our study is in keeping with published results [18,19,20]. While the preparation method was not related to DSS in the current research, we demonstrate that rhTSH was associated with positive PFS outcomes. Specifically, we identified a group consisting of patients with RAI-avid metastases and prepared for RAI with rhTSH, who were more likely to have favorable long-term PFS. Based on our findings, the different PFS rates of patients in groups 1 and 2—who all had RAI-avid metastases—indicate that rhTSH preparation may have a positive impact on this survival endpoint. Therefore, preparation with rhTSH in patients with distant metastases from PT—especially with RAI-avid metastases—appears to have encouraging long-term survival results and warrants further investigation.

There are several limitations pertaining to the present study. Given the non-randomized retrospective design, selection bias cannot be excluded. Therefore, our results require prospective confirmation in larger studies to verify their generalizability. While all of the study participants were diagnosed with PTC, fourteen distinct histological subtypes of this malignancy have been described—some of these being biologically aggressive and portending a poor prognosis [21]. Unfortunately, we had no data concerning PTC subtypes; therefore, they could not be examined in a separate subgroup analysis. A total of 34 patients had regional lymph node metastases at the date of diagnosis, whereas 26 did not. The remaining 28 patients had missing data concerning this variable; in this scenario, a detailed statistical analysis was unfeasible. Furthermore, the treatment of patients with non-RAI-avid metastases during the course of the disease was not standardized. Sorafenib and lenvatinib can improve PFS in patients with non-RAI-avid DTC [22,23], but their use in our study was not randomized and was left to the physicians’ discretion. Finally, preparation with rhTSH stimulation is not financially covered by the Taiwan public health insurance system. Therefore, patients in this arm incurred extra costs compared with those who received the preparation with THW. The increased financial burden posed by rhTSH may have resulted in missed dosing, at least for some patients, although this potential source of confounding was not specifically addressed in our study.

## 5. Conclusions

Preparation for RAI therapy using rhTSH stimulation is safe and feasible in patients with distant metastases from PTC, without compromising long-term survival outcomes. Specifically, we identified a group consisting of patients with RAI-avid metastases and prepared for RAI with rhTSH, who were more likely to have favorable long-term PFS. Our results require prospective confirmation in larger studies to verify their generalizability.

## Figures and Tables

**Table 1:** General characteristics of patients with metastatic papillary thyroid cancer according to the preparation method.

	THW (*n* = 51)	rhTSH (*n* = 37)	*p*-Value
Age at diagnosis, *n* (%)			
<55 years	38 (75%)	20 (54%)	0.068
≥55 years	13 (25%)	17 (46%)	
Sex, *n* (%)			
Man	23 (45%)	13 (35%)	0.386
Woman	28 (55%)	24 (65%)	
Site of distant metastasis, *n* (%)			
Isolated pulmonary metastases	37 (73%)	25 (68%)	0.642
Other sites ^1^	14 (27%)	12 (32%)	
Diagnostic time of distant metastasis, *n* (%)			
Initial	25 (49%)	19 (51%)	1.000
Late	26 (51%)	18 (49%)	
Stage at diagnosis ^2^, *n* (%)			
I–II	35 (69%)	21 (57%)	0.271
III–IV	16 (31%)	16 (43%)	
Cycles of RAI therapy, median (range)	6 (1–16)	5 (2–16)	0.769
RAI avidity, *n* (%)			
RAI avid	38 (75%)	26 (70%)	0.809
RAI non-avid	13 (25%)	11 (30%)	
Targeted therapy, *n* (%)			
No	41 (80%)	28 (76%)	0.610
Yes	10 (20%)	9 (24%)	

THW thyroid hormone withdrawal, rhTSH recombinant human thyroid-stimulating hormone. ^1^ Metastases to other organs, independent of the presence of pulmonary lesions. ^2^ AJCC Cancer Staging Manual, Eighth Edition.

**Table 2:** Univariate and multivariate analyses of risk factors for progression-free survival.

	Univariate Analysis	*p*-Value	Multivariate Analysis	*p*-Value
	HR (95% CI)		**HR (95% CI)**	
Sex				
Woman vs. man	0.566 (0.305–1.049)	0.071		
Age at diagnosis				
<55 year vs. ≥55 year	0.741 (0.386–1.422)	0.367		
Preparation				
rhTSH vs. THW	0.551 (0.285–1.065)	0.076	0.499 (0.256―0.971)	0.041
Diagnostic time of distant metastasis				
Initial vs. late	0.892 (0.482–1.651)	0.716		
Site of distant metastasis				
Isolated pulmonary metastases vs. other sites ^1^	1.011 (0.515–1.985)	0.974		
Stage at diagnosis ^2^				
I + II vs. III + IV	0.657 (0.339–1.273)	0.214		
RAI avidity				
RAI-avid vs. Non-RAI-avid	0.539 (0.290–1.004)	0.052	0.488 (0.260―0.914)	0.025
Targeted therapy				
No vs. Yes	0.701 (0.362–1.356)	0.291		

HR: hazard ratio, CI: confidence interval. ^1^ Metastases to other organs, independent of the presence of pulmonary lesions. ^2^ AJCC Cancer Staging Manual, Eighth Edition.

**Table 3:** Univariate and multivariate analyses of risk factors for disease-specific survival.

	Univariate Analysis	*p*-Value	Multivariate Analysis	*p*-Value
	HR (95% CI)		HR (95% CI)	
Sex				
Woman vs. man	0.301 (0.114–0.795)	0.015	0.305 (0.115–0.808)	0.017
Age at diagnosis				
<55 year vs. ≥55 year	0.250 (0.099–0.627)	0.003	0.246 (0.098–0.617)	0.003
Preparation				
rhTSH vs. THW	0.814 (0.327–2.027)	0.658		
Diagnostic time of distant metastasis				
Initial vs. late	0.870 (0.349–2.172)	0.766		
Site of distant metastasis				
Isolated pulmonary metastases vs. other sites ^1^	0.430 (0.174–1.063)	0.068	0.383 (0.149–0.985)	0.047
Stage at diagnosis ^2^				
I + II vs. III + IV	0.335 (0.133–0.844)	0.020		
RAI avidity				
RAI-avid vs. Non-RAI-avid	0.410 (0.166–1.012)	0.053		
Targeted therapy				
No vs. yes	0.891 (0.337–2.358)	0.816		

HR: hazard ratio, CI: confidence interval. ^1^ Metastases to other organs, independent of the presence of pulmonary lesions. ^2^ AJCC Cancer Staging Manual, Eighth Edition.

## Data Availability

The datasets generated during and/or analyzed during the current study are available from the corresponding author on reasonable request.
